# New Appliances and Things Medical

**Published:** 1901-10-05

**Authors:** 


### NEW APPLIANCES AND THINGS MEDICAL.

[We shall be glad to receive, at our Office, 28 & 29 Southampton Street, Strand, London, W.C., from the manufacturers, specimens of all new preparations and appliances which may be brought out from time to time.]

### THE ALLENBURYS FEEDER.

(Allen and Hanburys, Ltd., Bethnal Green, London, E.)

The features of the Allenburys feeder are designed with a view to obviating the objections which experience has taught us can be urged against the majority of infant feeding bottles. Firstly, a short rubber nipple fits directly on to the end of the bottle, thus obviating the dangers which attach to long rubber tubes, and this nipple can be turned inside out, and thus effectually cleaned after use. The end of the bottle opposite the teat is fitted with a very simple and ingenious rubber valve which admits air, but will not allow of the escape of the bottle's contents in the opposite direction. The shape of the bottle is such as to permit of the level of the valve, when the bottle is held in the usual position, being above that of the contained milk, so that air can enter to replace the milk as it is sucked up by the infant, thus eliminating the possibility of a partial vacuum within the bottle. The feeder can be cleaned out most thoroughly by running water from the hot-water tap in at one end and allowing it to flow out of the other. In every respect this bottle is as nearly perfect as possible.

### WILSON'S PATENT "SAFETY" BRONCHITIS-KETTLE STAND.

(R. & W. Wilson and Sons, Limited, 90 Wardour Street, London, W.)

Anyone who in the drowsy hours of the night has had the misfortune to upset a bronchitis-kettle will appreciate this invention, the very obviousness of which makes one feel vexed that it had not been made before. There is no attempt here to introduce a new kettle, the well-known Allen's bronchitis-kettle, with its ventilating pipe, serving every useful purpose. What Messrs. Wilson have done is to provide a strong firmly-standing tin box, 26 inches high by 14 inches square, with proper shelf and ventilating lid, in which the kettle is so placed as to be protected from accident. It seems a little thing to have thought of, but it is of such trifles that success is made. To upset a bronchitis-kettle and have a flare up with the curtains of a tracheotomy case is no trifling matter, and a device like this which will eliminate such a risk is a thing which should be welcomed in any hospital.

